# Nonlinear Stability of Natural-Fiber-Reinforced Composite Cylindrical Shells with Initial Geometric Imperfection Considering Moisture Absorption and Hygrothermal Aging

**DOI:** 10.3390/ma15196917

**Published:** 2022-10-05

**Authors:** Hongyu Zhang, Haifeng Bai, Zhongyi Zuo

**Affiliations:** 1 School of Mechanical Engineering, Dalian Jiaotong University, Dalian 116028, China; 2 Dalian Rail Transit Design Institute Co., Ltd., Dalian 116021, China; 3 School of Civil Engineering, Dalian Jiaotong University, Dalian 116028, China; 4 School of Traffic and Transportation Engineering, Dalian Jiaotong University, Dalian 116028, China

**Keywords:** stability, natural fiber-reinforced composite, cylindrical shell, initial geometric imperfection, moisture absorption and hygrothermal aging

## Abstract

In this paper, the nonlinear stability of a natural-fiber-reinforced composite cylindrical shell with initial geometric imperfection is investigated. The nonlinear governing equations are established by high-order shear deformation theory. The load-edge shortening curves for different imperfection amplitudes are obtained by the Galerkin method. Several numerical examples are presented to verify the accuracy of the proposed method and to investigate the influence of initial geometric imperfection, moisture absorption, and hygrothermal aging on the post-buckling behavior of natural-fiber-reinforced composite cylindrical shells.

## 1. Introduction

Natural-fiber-reinforced composite (NFRC) is a kind of composite made of bio-based fibers and polymer matrix [1,2]. Compared with traditional glass/carbon fiber-reinforced composites, NFRC has many advantages, such as low cost, light weight, superior degradability, renewable, and so on [3]. NFRC is more environment friendly and has been widely used in railway coaches, automobiles, aerospace, buildings, construction, and protective armors [4,5]. However, NFRC still has the disadvantage of the significant degradation of mechanical properties caused by moisture absorption and hygrothermal aging [6,7]. This is because the natural fiber has a porous structure capable of absorbing and storing large amounts of water. Water molecules occupy the free spaces as free water and they also make connections with polymer chains as bound water. The main process of moisture absorption is the diffusion of water molecules into the micropores between polymer chains of natural fibers; the natural fibers swell dramatically and the mechanical properties of the natural fibers are significantly degraded. The second process is the capillary transport into the gaps and flaws generated at the interfaces between the fibers and the polymer matrix due to incomplete wettability and impregnation [8,9]. Therefore, it is of great significant to consider moisture absorption and hygrothermal aging in the investigation of mechanical behaviors of NFRC structures.

Some researchers have studied the mechanical properties of natural-fiber-reinforced composites. Khakpour et al. [10] investigated the effects of fiber treatment, fiber length, and fiber density on the mechanical properties of structural adhesives enhanced with natural date palm tree fibers. Akhavan-Safar et al. [11] investigated the effect of natural date palm tree fibers (DPTF) on the R-curve of E-glass/epoxy end-notch-flexure samples. Dalla Libera et al. [12] manufactured prepregs by impregnating discontinuous curaua fibers with B-stage epoxy resin and investigated the thermal, dynamic mechanical, mechanical, and morphological behavior. Ramaswamy et al. [13] provided the analytical formulation to calculate the free-edge stress values induced in natural fiber composite materials. Pawar et al. [14] studied the dynamic and mechanical characteristics of banana-epoxy, kenaf-epoxy, and hemp-epoxy natural-fiber-reinforced composite materials. Nandhakumar et al. [15] performed the experimental investigation of the mechanical properties (rigidity and hardness quality) of natural-fiber-reinforced composites. Patel and Jain [16] studied the mechanical properties in randomly oriented short natural fiber composites and discussed the different combinations of short natural fibers with reinforced plastics. Gupta et al. [17] investigated the effects of fiber orientation on the mechanical properties of natural-fiber-reinforced epoxy composite by a finite element method. The mechanical behaviors of NFRC beams, plates, and shells have also been investigated. Lim et al. [18] performed the direct measurement of the whole-field deformation of an NFRC cantilever beam by the shadow moiré method and the comparison of the experimental results with the finite element results. Saravana Bavan and Mohan Kumar [19] analyzed the deflection and stress properties of an NFRC beam by the finite element method (FEM). Jirawattanasomkul et al. [20] investigated the shear behavior of pre-damaged deep reinforced concrete beams strengthened with NFRC sheets by the non-reversed cyclic three-point bending load test. Rajeshkumar and Hariharan [21] investigated the free vibration of NFRC beams for different fiber gauge lengths under clamped-free boundary condition. Rajesh et al. [22] investigated the influence of surface pre-treatment with sodium hydroxide and hybridization effect on the free vibration of NFRC beams. Senthil Kumar et al. [23] conducted an experimental investigation on the free vibration of NFPC beams and studied the influence of fiber length and weight percentage on the mechanical properties and free vibration characteristics. Kuriakose et al. [24] investigated the free vibration of NFRC plates and analyzed the influence of carbon nanotube fillers on the vibration behavior. Selvaraj et al. [25] investigated the material, mechanical, and dynamic characteristics of natural-fiber-reinforced composite sandwich plates with multiple-core layers. Wang and Wang [26] proposed a mechanical model for NFRC under hydrothermal loading and obtained the closed-form solutions for the buckling load and natural frequency of simply supported NFRC plates. Xu et al. [27] performed the free vibration and buckling analysis of a partially or internally cracked NFRC plate with corner-point supports by using the symplectic method. Zhang et al. [28] investigated the buckling of NFRC cylindrical shells based on Reissner’s shell theory and the symplectic method.

From the above research, it can be found that there have been few investigations into NFRC structures, especially the NFRC cylindrical shell. As an important basic structure in engineering, the cylindrical shell has the advantages of light weight, strong bearing capacity, and simple processing, and the main failure form is buckling instability [29]. The existing research on the stability of NFRC cylindrical shell [28] is based on the eigenvalue buckling of a perfect shell, and experimental research [30] found that buckling instability will occur under a load lower than the theoretical critical load. Koiter [31] pointed out that this is because the actual structure inevitably has initial geometric imperfection that will significantly reduce the stability. The stability of cylindrical shells considering the initial geometric imperfection has been investigated by some researchers. Narayana et al. [32] investigated the post-buckling and imperfection sensitivity of isotropic and composite cylindrical shells by FEM. Sun et al. [33] proposed the accelerated Koiter method to analyze the post-buckling and imperfection sensitivity of isotropic, composite and stiffened cylindrical shells. Shariyat and Eslami [34] and Eslami et al. [35] investigated the dynamic post-buckling of imperfect orthotropic and laminated cylindrical shells by using the finite difference method and Runge–Kutta methods. Shen [36,37,38,39,40] proposed the boundary layer theory and analyzed the post-buckling behavior of imperfect composite cylindrical shells by using the singular perturbation technique. Using a similar method, Babaei et al. [41] investigated the post-buckling of imperfect functionally graded material (FGM) cylindrical shells. Li et al. [42] analyzed the post-buckling of imperfect anisotropic laminated cylindrical shell. Foroutan et al. [43] analyzed the static and dynamic post-buckling of imperfect stiffened FGM cylindrical shells by the Galerkin method and Runge–Kutta methods. It can be seen that the research into the stability of imperfect cylindrical shells mainly depends on numerical methods; there is little analytical research.

Motivated by these reasons, we proposed an analytical method for the post-buckling of imperfect NFRC cylindrical shells with moisture absorption and hygrothermal aging. Firstly, the nonlinear governing equations for the post-buckling of imperfect NFRC cylindrical shells are established based on higher-order shear deformation theory. Secondly, the displacement functions and imperfection function are proposed and the load-edge shortening curves are obtained by the Galerkin method. Finally, the accuracy is verified and the influences of initial geometric imperfection, moisture absorption, and hygrothermal aging are discussed.

This paper is organized as follows: Section 2 presents the model of imperfect NFRC cylindrical shells. Section 3 presents the basic equations for post-buckling problems. Section 4 presents the procedure to solve the post-buckling equilibrium path. Section 5 presents the numerical examples and discusses the effects of calculation parameters. The conclusions are drawn in Section 6.

## 2. Model of Imperfect NFRC Cylindrical Shells

**Figure 1:**
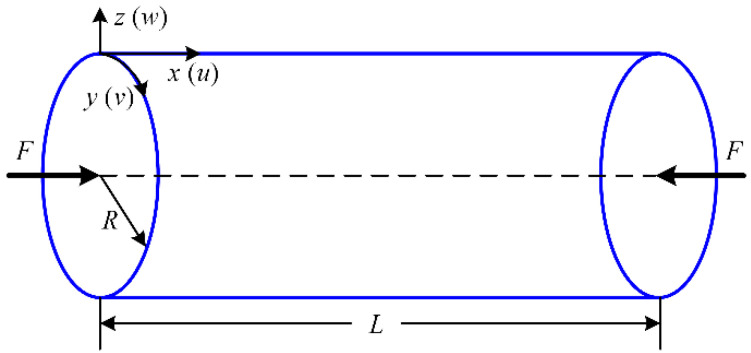
Schematic diagram of NFRC cylindrical shell.

Consider an imperfect NFRC cylindrical shell under axial compression *F* in Figure 1. The composite is unidirectional long sisal fiber-reinforced benzylated wood. The layer number of the NFRC cylindrical shell is 8 and the layer angle is (0/90)_2S_. The geometric parameters of the shell are taken as length *L*, radius of the mid-surface *R*, and thickness *h*. It refers to an orthogonal curvilinear coordinate system in which the *x−*, *y−*, and *z−* axes represent the longitudinal, circumferential, and normal of the mid-surface of the shell, respectively. The displacements of the mid-surface of the shell along the *x−*, *y−*, and *z−* axes are *u*, *v*, and *w*, respectively. *Φx* and *Φy* are the rotations of the transverse normal.

The effect of the material properties of natural-fiber-reinforced composite considering the influence of moisture absorption and hygrothermal aging can be expressed as [26,27]:

(1) $$E_{1}=\frac{3G\left(1-v_{f}^{2}\beta_{s}\gamma_{s}-v_{f}^{3}\beta_{l}\gamma_{l}\right)+4v_{f}\eta G\left(1-\chi_{s}\gamma_{s}\right)\left(c^{4}+s^{4}-c^{2}s^{2}\right)}{1+v_{f}\eta \left[\left(1-\chi_{s}\gamma_{s}\right)/\left(1-v_{f}^{2}\beta_{s}\gamma_{s}-v_{f}^{3}\beta_{l}\gamma_{l}\right)\right]s^{4}}$$

(2) $$E_{2}=\frac{3G\left(1-v_{f}^{2}\beta_{s}\gamma_{s}-v_{f}^{3}\beta_{l}\gamma_{l}\right)+4v_{f}\eta G\left(1-\chi_{s}\gamma_{s}\right)\left(c^{4}+s^{4}-c^{2}s^{2}\right)}{1+v_{f}\eta \left[\left(1-\chi_{s}\gamma_{s}\right)/\left(1-v_{f}^{2}\beta_{s}\gamma_{s}-v_{f}^{3}\beta_{l}\gamma_{l}\right)\right]c^{4}}$$

(3) $$\nu_{12}=\frac{\left(1-v_{f}^{2}\beta_{s}\gamma_{s}-v_{f}^{3}\beta_{l}\gamma_{l}\right)+2v_{f}\eta \left(1-\chi_{s}\gamma_{s}\right)c^{2}s^{2}}{2\left(1-v_{f}^{2}\beta_{s}\gamma_{s}-v_{f}^{3}\beta_{l}\gamma_{l}\right)+2v_{f}\eta \left(1-\chi_{s}\gamma_{s}\right)s^{4}}$$

(4) $$\nu_{21}=\frac{\left(1-v_{f}^{2}\beta_{s}\gamma_{s}-v_{f}^{3}\beta_{l}\gamma_{l}\right)+2v_{f}\eta \left(1-\chi_{s}\gamma_{s}\right)c^{2}s^{2}}{2\left(1-v_{f}^{2}\beta_{s}\gamma_{s}-v_{f}^{3}\beta_{l}\gamma_{l}\right)+2v_{f}\eta \left(1-\chi_{s}\gamma_{s}\right)c^{4}}$$

(5) $$G_{12}=G\left(1-v_{f}^{2}\beta_{s}\gamma_{s}-v_{f}^{3}\beta_{l}\gamma_{l}\right)+4v_{f}\eta G\left(1-\chi_{s}\gamma_{s}\right)c^{2}s^{2}$$

(6) $$G_{13}=G_{23}=G\left(1-v_{f}^{2}\beta_{s}\gamma_{s}-v_{f}^{3}\beta_{l}\gamma_{l}\right)$$

where *E*_1_, *E*_2_, *G*_12_, *G*_13_, *G*_23_, and *ν*_12_, *ν*_21_ are the Young’s moduli, shear moduli, and Poisson’s ratio, respectively, *c* = cos*θ*, *s* = sin*θ* and *θ* is the layer angle, *γ_s_* and *γ_l_* are two important coefficients describing the damage evolution induced by moisture absorption and hygrothermal aging and their expressions are $\gamma_{s}=1-e^{-\eta v_{f}Gt/D}$ and $\gamma_{l}=1-e^{-v_{f}Tt}-v_{f}Tte^{-v_{f}Tt}$, *v_f_* is the fiber content, *η* is a dimensionless constant which can be determined by fitting the experiential date, *D* is a material parameter which governing the speed of energy dissipation, *G* is the initial shear modulus of the composites, *t* is the aging time (in h for hours), *T* is a material index which can be determined by fitting the empirical data, and *β_s_*, *β_l_*, and *χ_s_* are three constants that can be obtained by three times multinomial fittings of the experimental results. These parameters are related to the experimental conditions (temperature, humidity, etc.). In this paper, we aim to propose an analytical method to analyze the post-buckling of imperfect NFRC cylindrical shells, so we select only the values available in the open literature [26] under specific humidity and temperature; the parameters are *G* = 0.104 GPa, *η* = 90.870, *β_s_* = 7.3459, *β_l_* = 200.0104, *χ_s_* = 0.6171, *D* = 56.18 hN/m^2^, and *T* = 0.1/h.

## 3. Basic Equations for Post-Buckling of Imperfect NFRC Cylindrical Shells

According to the high-order shear deformation theory [44], the displacement components at a point of the shell can be expressed as:

(7) $$u_{1}\left(x,\;y,\;z\right)=u\left(x,\;y\right)-\frac{4z^{3}}{3h^{2}}\frac{\partial w\left(x,\;y\right)}{\partial x}+\left(z-\frac{4z^{3}}{3h^{2}}\right)\Phi_{x}\left(x,\;y\right)$$

(8) $$u_{2}\left(x,\;y,\;z\right)=\left(1+\frac{z}{R}\right)v\left(x,\;y\right)-\frac{4z^{3}}{3h^{2}}\frac{\partial w\left(x,\;y\right)}{\partial y}+\left(z-\frac{4z^{3}}{3h^{2}}\right)\Phi_{y}\left(x,\;y\right)$$

(9) $$u_{3}\left(x,\;y,\;z\right)=w\left(x,\;y\right)+w^{0}\left(x,\;y\right)$$

Based on Novozhilov’s nonlinear shell theory [45], the nonlinear strain–displacement relations are:

(10) $$\varepsilon_{x}=\frac{\partial u_{1}}{\partial x}+\frac{1}{2}\left[{\left(\frac{\partial u_{1}}{\partial x}\right)}^{2}+{\left(\frac{\partial u_{2}}{\partial x}\right)}^{2}+{\left(\frac{\partial u_{3}}{\partial x}\right)}^{2}\right]$$

(11) $$\varepsilon_{y}=\frac{\frac{\partial u_{2}}{\partial y}+\frac{u_{3}}{R}}{1+\frac{z}{R}}+\frac{{\left(\frac{\partial u_{1}}{\partial y}\right)}^{2}+{\left(\frac{\partial u_{2}}{\partial y}+\frac{u_{3}}{R}\right)}^{2}+{\left(\frac{u_{2}}{R}-\frac{\partial u_{3}}{\partial y}\right)}^{2}}{2{\left(1+\frac{z}{R}\right)}^{2}}$$

(12) $$\gamma_{xy}=\frac{\partial u_{2}}{\partial x}+\frac{\frac{\partial u_{1}}{\partial y}}{1+\frac{z}{R}}+\frac{\partial u_{1}}{\partial x}\frac{\partial u_{1}}{\partial y}+\frac{\partial u_{2}}{\partial x}\left(\frac{\partial u_{2}}{\partial y}+\frac{u_{3}}{R}\right)+\frac{\partial u_{3}}{\partial x}\left(\frac{u_{2}}{R}-\frac{\partial u_{3}}{\partial y}\right)$$

(13) $$\gamma_{xz}=\frac{\partial u_{1}}{\partial z}+\frac{\partial u_{3}}{\partial x}+\frac{\partial u_{1}}{\partial x}\frac{\partial u_{1}}{\partial z}+\frac{\partial u_{2}}{\partial x}\frac{\partial u_{2}}{\partial z}+\frac{\partial u_{3}}{\partial x}\frac{\partial u_{3}}{\partial z}$$

(14) $$\gamma_{yz}=\frac{\partial u_{2}}{\partial z}+\frac{\frac{\partial u_{3}}{\partial y}-\frac{u_{2}}{R}}{1+\frac{z}{R}}+\frac{\frac{\partial u_{1}}{\partial y}\frac{\partial u_{1}}{\partial z}+\left(\frac{\partial u_{2}}{\partial y}+\frac{u_{3}}{R}\right)\frac{\partial u_{2}}{\partial z}+\left(\frac{\partial u_{3}}{\partial y}-\frac{u_{2}}{R}\right)\frac{\partial u_{3}}{\partial z}}{1+\frac{z}{R}}$$

Substituting Equations (7)–(9) into Equations (10)–(14) and retaining only the nonlinear terms with *w* and *w*^0^, we have:

(15) $$\varepsilon =\varepsilon^{\left(0\right)}+z\chi^{\left(0\right)}+z^{3}\chi^{\left(2\right)}$$

(16) $$\gamma =\gamma^{\left(0\right)}+z^{2}\chi^{\left(1\right)}$$

where $\varepsilon ={\left\{\varepsilon_{x},\varepsilon_{y},\gamma_{xy}\right\}}^{T}$, $\gamma ={\left\{\gamma_{xz},\gamma_{yz}\right\}}^{T}$, and:

(17) $$\varepsilon^{\left(0\right)}=\left\{\begin{array}{c} \varepsilon_{x}^{\left(0\right)}\\ \varepsilon_{y}^{\left(0\right)}\\ \gamma_{xy}^{\left(0\right)} \end{array}\right\}=\left\{\begin{array}{c} \frac{\partial u}{\partial x}+\frac{1}{2}{\left(\frac{\partial w}{\partial x}\right)}^{2}+\frac{\partial w}{\partial x}\frac{\partial w^{0}}{\partial x}\\ \frac{\partial v}{\partial y}+\frac{w}{R}+\frac{1}{2}{\left(\frac{\partial w}{\partial y}\right)}^{2}+\frac{\partial w}{\partial y}\frac{\partial w^{0}}{\partial y}\\ \frac{\partial u}{\partial y}+\frac{\partial v}{\partial x}+\frac{\partial w}{\partial x}\frac{\partial w}{\partial y}+\frac{\partial w}{\partial x}\frac{\partial w^{0}}{\partial y}+\frac{\partial w^{0}}{\partial x}\frac{\partial w}{\partial y} \end{array}\right\}$$

(18) $$\chi^{\left(0\right)}=\left\{\begin{array}{c} \chi_{x}^{\left(0\right)}\\ \chi_{y}^{\left(0\right)}\\ \chi_{xy}^{\left(0\right)} \end{array}\right\}=\left\{\begin{array}{c} \frac{\partial \Phi_{x}}{\partial x}\\ \frac{\partial \Phi_{y}}{\partial y}\\ \frac{\partial \Phi_{x}}{\partial y}+\frac{\partial \Phi_{y}}{\partial x} \end{array}\right\}$$

(19) $$\chi^{\left(2\right)}=\left\{\begin{array}{c} \chi_{x}^{\left(2\right)}\\ \chi_{y}^{\left(2\right)}\\ \chi_{xy}^{\left(2\right)} \end{array}\right\}=-\frac{4}{3h^{2}}\left\{\begin{array}{c} \frac{\partial \Phi_{x}}{\partial x}+\frac{\partial^{2}w}{\partial x^{2}}\\ \frac{\partial \Phi_{y}}{\partial y}+\frac{\partial^{2}w}{\partial y^{2}}\\ \frac{\partial \Phi_{x}}{\partial y}+\frac{\partial \Phi_{y}}{\partial x}+\frac{\partial^{2}w}{\partial x\partial y} \end{array}\right\}$$

(20) $$\gamma^{\left(0\right)}=\left\{\begin{array}{c} \gamma_{xz}^{\left(0\right)}\\ \gamma_{yz}^{\left(0\right)} \end{array}\right\}=\left\{\begin{array}{c} \Phi_{x}+\frac{\partial w}{\partial x}\\ \Phi_{y}+\frac{\partial w}{\partial y} \end{array}\right\}$$

(21) $$\chi^{\left(1\right)}=\left\{\begin{array}{c} \chi_{xz}^{\left(1\right)}\\ \chi_{yz}^{\left(1\right)} \end{array}\right\}=-\frac{4}{h^{2}}\left\{\begin{array}{c} \Phi_{x}+\frac{\partial w}{\partial x}\\ \Phi_{y}+\frac{\partial w}{\partial y} \end{array}\right\}$$

The constitutive equation in the *k*-th lamina of the imperfect NFRC cylindrical shell can be written as:

(22) $$\left\{\begin{array}{c} \sigma_{x}^{k}\\ \sigma_{y}^{k}\\ \tau_{xy}^{k}\\ \tau_{xz}^{k}\\ \tau_{yz}^{k} \end{array}\right\}=\left[\begin{array}{ccccc} C_{11}^{k} & C_{12}^{k} & 0 & 0 & 0\\ C_{21}^{k} & C_{22}^{k} & 0 & 0 & 0\\ 0 & 0 & G_{12}^{k} & 0 & 0\\ 0 & 0 & 0 & G_{13}^{k} & 0\\ 0 & 0 & 0 & 0 & G_{23}^{k} \end{array}\right]\left\{\begin{array}{c} \varepsilon_{x}^{k}\\ \varepsilon_{y}^{k}\\ \gamma_{xy}^{k}\\ \gamma_{xz}^{k}\\ \gamma_{yz}^{k} \end{array}\right\}$$

where $\sigma_{x}^{k}$, $\sigma_{y}^{k}$, $\tau_{xy}^{k}$, $\tau_{xz}^{k}$, and $\tau_{yz}^{k}$ are the in-plane stress and transverse shear stress. $C_{11}^{k}=E_{1}^{k}/\left(1-\nu_{12}^{k}\nu_{21}^{k}\right)$, $C_{12}^{k}=C_{21}^{k}=E_{1}^{k}\nu_{21}^{k}/\left(1-\nu_{12}^{k}\nu_{21}^{k}\right)$, and $C_{22}^{k}=E_{2}^{k}/\left(1-\nu_{12}^{k}\nu_{21}^{k}\right)$.

Integrating Equation (14) across the thickness, the resultant force vector **N**, moment vector **M**, high-order force vector **P**, shear force vector **Q,** and high-order shear force vector **R** can be obtained as:

(23) $$N={\left\{N_{x},\;N_{y},\;N_{xy}\right\}}^{T}=\sum_{i=1}^{K}\int_{h_{i}}^{h_{i+1}}{\left\{\sigma_{x}^{k},\;\sigma_{y}^{k},\;\tau_{xy}^{k}\right\}}^{T}\;dz$$

(24) $$M={\left\{M_{x},\;M_{y},\;M_{xy}\right\}}^{T}=\sum_{i=1}^{K}\int_{h_{i}}^{h_{i+1}}{\left\{\sigma_{x}^{k},\;\sigma_{y}^{k},\;\tau_{xy}^{k}\right\}}^{T}zdz$$

(25) $$P={\left\{P_{x},\;P_{y},\;P_{xy}\right\}}^{T}=\sum_{i=1}^{K}\int_{h_{i}}^{h_{i+1}}{\left\{\sigma_{x}^{k},\;\sigma_{y}^{k},\;\tau_{xy}^{k}\right\}}^{T}z^{3}dz$$

(26) $$Q={\left\{Q_{x},\;Q_{y}\right\}}^{T}=\sum_{i=1}^{K}\int_{h_{i}}^{h_{i+1}}{\left\{\tau_{xz}^{k},\;\tau_{yz}^{k}\right\}}^{T}dz$$

(27) $$R={\left\{R_{x},\;R_{y}\right\}}^{T}=\sum_{i=1}^{K}\int_{h_{i}}^{h_{i+1}}{\left\{\tau_{xz}^{k},\;\tau_{yz}^{k}\right\}}^{T}z^{2}dz$$

where *K* is the layer number.

The equilibrium equations of imperfect NFRC cylindrical shells can be expressed as:

(28) $$\frac{\partial N_{x}}{\partial x}+\frac{\partial N_{xy}}{\partial y}=0$$

(29) $$\frac{\partial N_{xy}}{\partial x}+\frac{\partial N_{y}}{\partial y}=0$$

(30) $$\begin{array}{l} \frac{\partial Q_{x}}{\partial x}+\frac{\partial Q_{y}}{\partial y}-\frac{4}{h^{2}}\left(\frac{\partial R_{x}}{\partial x}+\frac{\partial R_{y}}{\partial y}\right)+\frac{4}{3h^{2}}\left(\frac{\partial^{2}P_{x}}{\partial x^{2}}+2\frac{\partial^{2}P_{xy}}{\partial x\partial y}+\frac{\partial^{2}P_{y}}{\partial y^{2}}\right)\\ -\frac{N_{y}}{R}+N_{x}\frac{\partial^{2}\left(w+w^{0}\right)}{\partial x^{2}}+2N_{xy}\frac{\partial^{2}\left(w+w^{0}\right)}{\partial x\partial y}+N_{y}\frac{\partial^{2}\left(w+w^{0}\right)}{\partial y^{2}}=0 \end{array}$$

(31) $$\frac{\partial M_{x}}{\partial x}+\frac{\partial M_{xy}}{\partial y}-\frac{4}{3h^{2}}\left(\frac{\partial P_{x}}{\partial x}+\frac{\partial P_{xy}}{\partial y}\right)-Q_{x}+\frac{4}{h^{2}}R_{x}=0$$

(32) $$\frac{\partial M_{xy}}{\partial x}+\frac{\partial M_{y}}{\partial y}-\frac{4}{3h^{2}}\left(\frac{\partial P_{xy}}{\partial x}+\frac{\partial P_{y}}{\partial y}\right)-Q_{y}+\frac{4}{h^{2}}R_{y}=0$$

where *w*^0^ is the initial geometric imperfection.

Airy’s stress function *ψ* is introduced to simplify the equilibrium equations, which satisfy:

(33) $$N_{x}=\frac{\partial^{2}\psi }{\partial y^{2}},\;N_{y}=\frac{\partial^{2}\psi }{\partial x^{2}},\;N_{xy}=-\frac{\partial^{2}\psi }{\partial x\partial y}$$

The first two equilibrium equations, Equations (20) and (21), are transformed into a compatible equation as follows:

(34) $$\begin{array}{l} \frac{\partial^{2}\varepsilon_{x}^{\left(0\right)}}{\partial y^{2}}+\frac{\partial^{2}\varepsilon_{y}^{\left(0\right)}}{\partial x^{2}}-\frac{\partial^{2}\gamma_{xy}^{\left(0\right)}}{\partial x\partial y}=\frac{1}{R}\frac{\partial^{2}w}{\partial x^{2}}-\frac{\partial^{2}w}{\partial x^{2}}\frac{\partial^{2}w}{\partial y^{2}}\\ +{\left(\frac{\partial^{2}w}{\partial x\partial y}\right)}^{2}-\frac{\partial^{2}w}{\partial x^{2}}\frac{\partial^{2}w^{0}}{\partial y^{2}}+2\frac{\partial^{2}w}{\partial x\partial y}\frac{\partial^{2}w^{0}}{\partial x\partial y}-\frac{\partial^{2}w}{\partial y^{2}}\frac{\partial^{2}w^{0}}{\partial x^{2}} \end{array}$$

The equilibrium equations about stress function *ψ*, deflection *w*, and rotations of the transverse normal *Φx* and *Φy* can be rewritten as:

(35) $$\begin{array}{l} \delta_{1}\frac{\partial^{4}\psi }{\partial x^{4}}+\delta_{2}\frac{\partial^{4}\psi }{\partial x^{2}\partial y^{2}}+\delta_{3}\frac{\partial^{4}\psi }{\partial y^{4}}+\delta_{4}\frac{\partial^{4}w}{\partial x^{4}}+\delta_{5}\frac{\partial^{4}w}{\partial x^{2}\partial y^{2}}+\delta_{6}\frac{\partial^{4}w}{\partial y^{4}}\\ +\delta_{7}\frac{\partial^{3}\Phi_{x}}{\partial x^{3}}+\delta_{8}\frac{\partial^{3}\Phi_{x}}{\partial x\partial y^{2}}+\delta_{9}\frac{\partial^{3}\Phi_{y}}{\partial x^{2}\partial y}+\delta_{10}\frac{\partial^{3}\Phi_{y}}{\partial y^{3}}-\frac{1}{R}\frac{\partial^{2}w}{\partial x^{2}}+\frac{\partial^{2}w}{\partial x^{2}}\frac{\partial^{2}w}{\partial y^{2}}\\ -{\left(\frac{\partial^{2}w}{\partial x\partial y}\right)}^{2}+\frac{\partial^{2}w}{\partial x^{2}}\frac{\partial^{2}w^{0}}{\partial y^{2}}-2\frac{\partial^{2}w}{\partial x\partial y}\frac{\partial^{2}w^{0}}{\partial x\partial y}+\frac{\partial^{2}w}{\partial y^{2}}\frac{\partial^{2}w^{0}}{\partial x^{2}}=0 \end{array}$$

(36) $$\begin{array}{l} \delta_{4}\frac{\partial^{4}\psi }{\partial x^{4}}+\delta_{5}\frac{\partial^{4}\psi }{\partial x^{2}\partial y^{2}}+\delta_{6}\frac{\partial^{4}\psi }{\partial y^{4}}+\delta_{11}\frac{\partial^{4}w}{\partial x^{4}}+\delta_{12}\frac{\partial^{4}w}{\partial x^{2}\partial y^{2}}+\delta_{13}\frac{\partial^{4}w}{\partial y^{4}}+\delta_{14}\frac{\partial^{2}w}{\partial x^{2}}+\delta_{14}\frac{\partial^{2}w}{\partial y^{2}}\\ +\delta_{14}\frac{\partial \Phi_{x}}{\partial x}+\delta_{14}\frac{\partial \Phi_{y}}{\partial y}+\delta_{15}\frac{\partial^{3}\Phi_{x}}{\partial x^{3}}+\delta_{16}\frac{\partial^{3}\Phi_{x}}{\partial x\partial y^{2}}+\delta_{17}\frac{\partial^{3}\Phi_{y}}{\partial x^{2}\partial y}+\delta_{18}\frac{\partial^{3}\Phi_{y}}{\partial y^{3}}-\frac{1}{R}\frac{\partial^{2}\psi }{\partial x^{2}}\\ +\frac{\partial^{2}\psi }{\partial y^{2}}\frac{\partial^{2}w}{\partial x^{2}}-2\frac{\partial^{2}\psi }{\partial x\partial y}\frac{\partial^{2}w}{\partial x\partial y}+\frac{\partial^{2}\psi }{\partial x^{2}}\frac{\partial^{2}w}{\partial y^{2}}+\frac{\partial^{2}\psi }{\partial y^{2}}\frac{\partial^{2}w^{0}}{\partial x^{2}}-2\frac{\partial^{2}\psi }{\partial x\partial y}\frac{\partial^{2}w^{0}}{\partial x\partial y}+\frac{\partial^{2}\psi }{\partial x^{2}}\frac{\partial^{2}w^{0}}{\partial y^{2}}=0 \end{array}$$

(37) $$\begin{array}{l} \delta_{7}\frac{\partial^{3}\psi }{\partial x^{3}}+\delta_{8}\frac{\partial^{3}\psi }{\partial x\partial y^{2}}+\delta_{14}\frac{\partial w}{\partial x}+\delta_{15}\frac{\partial^{3}w}{\partial x^{3}}+\delta_{16}\frac{\partial^{3}w}{\partial x\partial y^{2}}\\ +\delta_{14}\Phi_{x}+\delta_{19}\frac{\partial^{2}\Phi_{x}}{\partial x^{2}}+\delta_{20}\frac{\partial^{2}\Phi_{x}}{\partial y^{2}}+\delta_{21}\frac{\partial^{2}\Phi_{y}}{\partial x\partial y}=0 \end{array}$$

(38) $$\begin{array}{l} \delta_{9}\frac{\partial^{3}\psi }{\partial x^{2}\partial y}+\delta_{10}\frac{\partial^{3}\psi }{\partial y^{3}}+\delta_{14}\frac{\partial w}{\partial y}+\delta_{16}\frac{\partial^{3}w}{\partial x^{2}\partial y}+\delta_{17}\frac{\partial^{3}w}{\partial y^{3}}\\ +\delta_{14}\Phi_{y}+\delta_{20}\frac{\partial^{2}\Phi_{y}}{\partial x^{2}}+\delta_{22}\frac{\partial^{2}\Phi_{y}}{\partial y^{2}}+\delta_{21}\frac{\partial^{2}\Phi_{x}}{\partial x\partial y}=0 \end{array}$$

The boundary conditions at the two ends of imperfect NFRC cylindrical shells are constrained and satisfy:

(39) $$w=0,\;\frac{\partial w}{\partial x}=0,\Phi_{x}=0,\;\Phi_{y}=0,\;\int_{0}^{2\pi R}N_{x}dy=-F$$

## 4. Procedure for Solving the Post-Buckling Equilibrium Path

The post-buckling equilibrium path could be solved by the Galerkin method, for which the trial functions needs to be assumed. The deformation for the post-buckling is very complex and the displacement function should have more expansion terms. The displacement functions that satisfy boundary condition (39) are assumed as [46,47]:

(40) $$w=\sum_{n}\sum_{m}a_{mn}\left\{\cos\left[\frac{\left(m-1\right)\pi x}{L}\right]-\cos\left[\frac{\left(m+1\right)\pi x}{L}\right]\right\}\cos\left(\frac{nyN}{R}\right)$$

(41) $$\Phi_{x}=\sum_{n}\sum_{m}b_{mn}\left\{\sin\left[\frac{\left(m-1\right)\pi x}{L}\right]-\sin\left[\frac{\left(m+1\right)\pi x}{L}\right]\right\}\cos\left(\frac{nyN}{R}\right)$$

(42) $$\Phi_{y}=\sum_{n}\sum_{m}c_{mn}\left\{\cos\left[\frac{\left(m-1\right)\pi x}{L}\right]-\cos\left[\frac{\left(m+1\right)\pi x}{L}\right]\right\}\sin\left(\frac{nyN}{R}\right)$$

where *N* is the circumferential wave number, *a_mn_*, *b_mn_*, and *c_mn_* are the undetermined coefficients; *m* and *n* are selected as *m* = 1, 3, 5… and *n* = 0, 1, 2.

The initial geometric imperfection function is assumed as [46]:

(43) $$w^{0}=0.5\mu h\left[1-\cos\left(\frac{2\pi x}{L}\right)\right]\cos\left(\frac{yN}{R}\right)$$

where *μ* is the imperfection amplitude.

Substituting Equations (40)–(43) into Equation (35), we have:

(44) $$\delta_{1}\frac{\partial^{4}\psi }{\partial x^{4}}+\delta_{2}\frac{\partial^{4}\psi }{\partial x^{2}\partial y^{2}}+\delta_{3}\frac{\partial^{4}\psi }{\partial y^{4}}=\sum_{p=0}\sum_{q=0}\psi_{pq}\cos\left(\frac{p\pi x}{L}\right)\;\cos\left(\frac{qyN}{R}\right)\;$$

The stress function can be expressed as a similar form to the right side of Equation (44) as follows:

(45) $$\psi =p_{x}y^{2}+\sum_{p=0}\sum_{q=0}\Psi_{pq}\cos\left(\frac{p\pi x}{L}\right)\;\cos\left(\frac{qyN}{R}\right)\;$$

where *p_x_* = −(*F*/2*π*^2^*R*^2^) is used to satisfy the load boundary condition.

To obtain the undetermined coefficients, the Galerkin method is applied to the equilibrium Equations (36)–(38), and the following relations can be obtained:

(46) $$\int_{0}^{2\pi R}\int_{0}^{L}L_{1}\left(w,\;\Phi_{x},\;\Phi_{y}\right)\left\{\cos\left[\frac{\left(m-1\right)\pi x}{L}\right]-\cos\left[\frac{\left(m+1\right)\pi x}{L}\right]\right\}\cos\left(\frac{nyN}{R}\right)dxdy=0$$

(47) $$\int_{0}^{2\pi R}\int_{0}^{L}L_{2}\left(w,\;\Phi_{x},\;\Phi_{y}\right)\left\{\sin\left[\frac{\left(m-1\right)\pi x}{L}\right]-\sin\left[\frac{\left(m+1\right)\pi x}{L}\right]\right\}\cos\left(\frac{nyN}{R}\right)dxdy=0$$

(48) $$\int_{0}^{2\pi R}\int_{0}^{L}L_{3}\left(w,\;\Phi_{x},\;\Phi_{y}\right)\left\{\cos\left[\frac{\left(m-1\right)\pi x}{L}\right]-\cos\left[\frac{\left(m+1\right)\pi x}{L}\right]\right\}\sin\left(\frac{nyN}{R}\right)dxdy=0$$

By solving the nonlinear equations presented in Equations (46)–(48), the undetermined coefficients *a_mn_*, *b_mn_*, and *c_mn_* can be determined and the end shortening can be calculated from the following equation:

(49) $$∆=-\int_{0}^{L}\frac{\partial u}{\partial x}\;dx$$

## 5. Numerical Results and Discussion

### 5.1. Comparison Study

**Figure 2:**
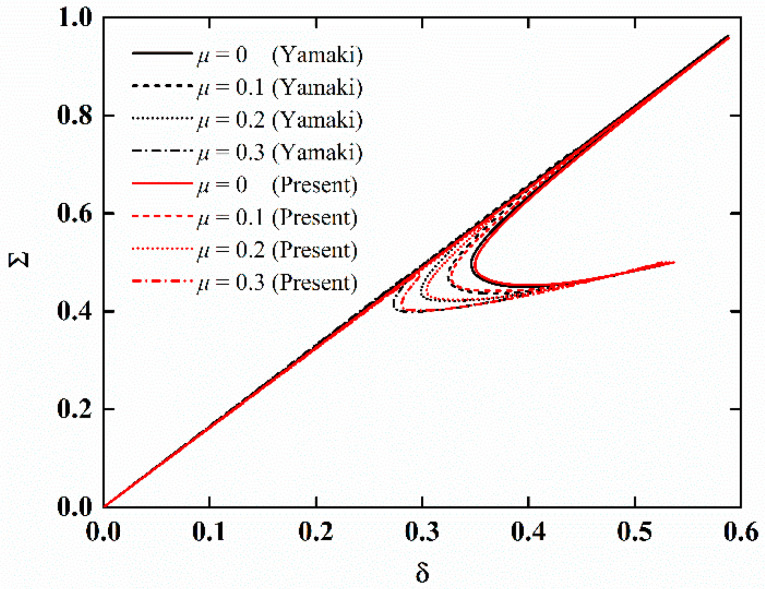
Comparison of load-edge shortening curves of imperfect isotropic cylindrical shells.

There is no available research on the stability of imperfect NFRC cylindrical shells in the open literature. Firstly, the present results are compared with those of imperfect isotropic cylindrical shells to verify the accuracy of the proposed method. The computation parameters are *E* = 5.56 GPa, *ν* = 0.3, *L* = 71.96 mm, *h* = 0.247 mm, and *R* = 100 mm. The dimensionless axial compression is *Σ* = *F*/*F_cl_* where *F_cl_* = 2*πEh*^2^/[3(1 *− ν*^2^)]^0.5^. The dimensionless edge shortening is *δ* = (*ΔR*)/(*Lh*). The load-edge shortening curves for different imperfection amplitudes are shown in Figure 2. It is obvious that the present results are in good agreement with the reference results [46].

**Figure 3:**
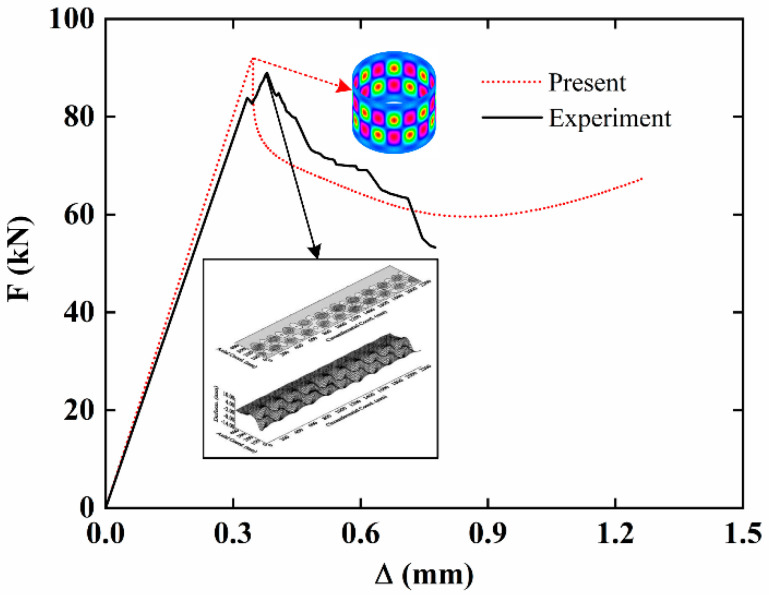
Comparison of load-edge shortening curves of imperfect carbon-fiber-reinforced composite cylindrical shells.

Subsequently, the present results are compared with the experimental results [48] for the buckling of imperfect carbon-fiber-reinforced composite cylindrical shells. The geometric parameters are *L* = 540 mm, *h* = 1.2 mm, *R* = 350 mm. The layer number is 8 and the laying angle is (0/90)_2S_. The load-edge shortening curves are shown in Figure 3. It is observed that the present results are in good agreement with the experimental results, and the buckling mode is very close.

**Figure 4:**
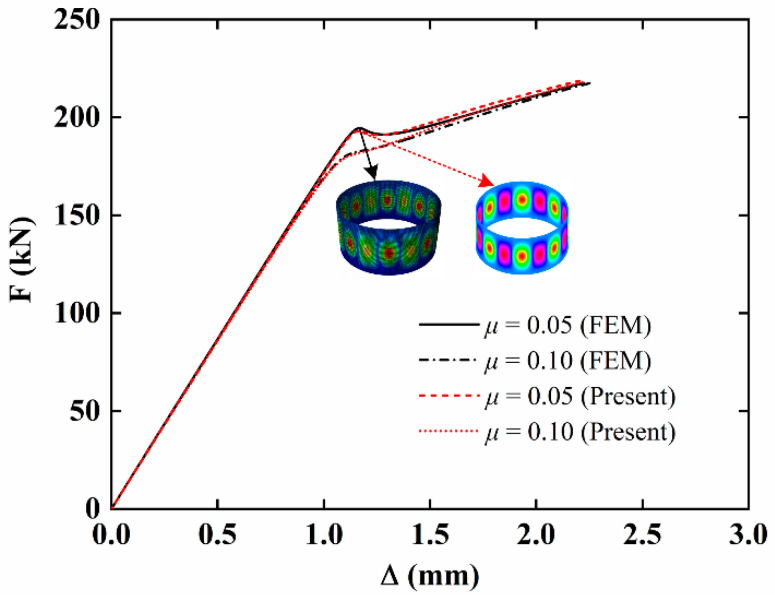
Comparison of load-edge shortening curves of single-layer imperfect NFRC cylindrical shells.

Finally, the present results are compared with the finite element (FE) results by considering single-layer imperfect NFRC cylindrical shells. The geometric parameters are *L* = 1000 mm, *h* = 10 mm, and *R* = 1000 mm. The laying angle is 0°, the fiber content is *v_f_* = 0.1, and the aging time is *t* = 50 h. The load-edge shortening curves for different imperfection amplitudes are presented in Figure 4. The good agreement with the FE results can also be observed. From the above comparison, it is deduced that the proposed method can accurately analyze the stability of imperfect NFRC cylindrical shells.

### 5.2. Parameter Study

In this section, the effects of initial geometric imperfection, moisture absorption and hygrothermal aging on the stability of NFRC cylindrical shells are investigated. The geometrical parameters are *L* = 100 mm, *h* = 1 mm, and *R* = 100 mm. The dimensionless axial compression is *Σ* = *F*/*F_cl_*, where *F_cl_* = 2*πE_m_h*^2^/[3(1 − *ν_m_*^2^)]^0.5^, *E_m_* = 0.312 GPa, and *ν_m_* = 0.5. The dimensionless edge shortening is *δ* = (*ΔR*)/(*Lh*).

**Figure 5:**
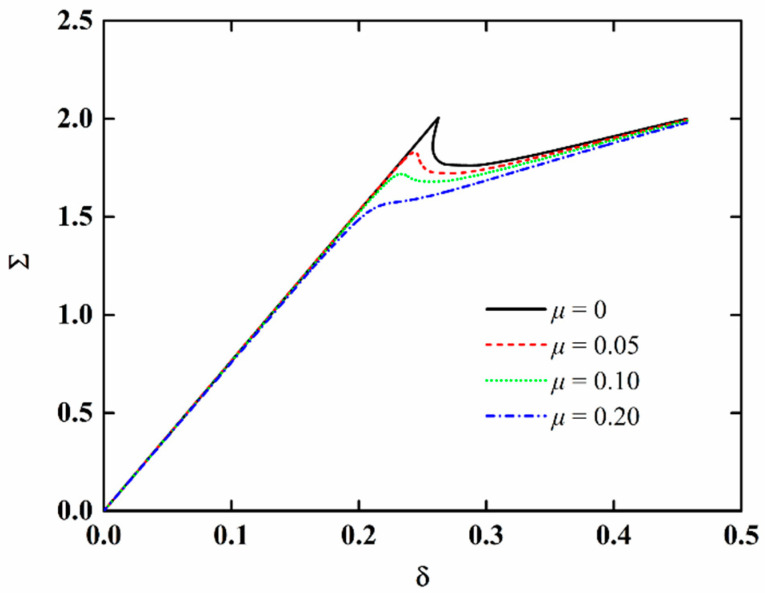
Load-edge shortening curves of imperfect NFRC cylindrical shells for different imperfection amplitudes.

**Figure 6:**
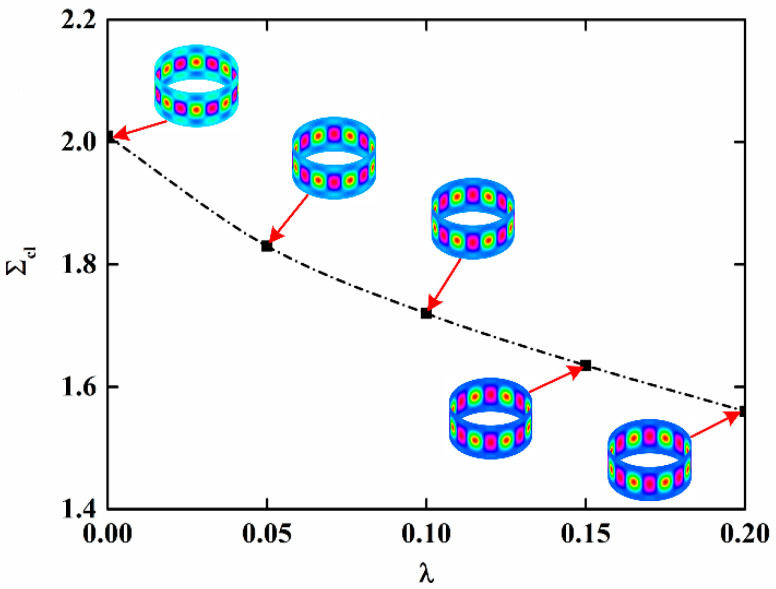
Variation in the critical load of imperfect NFRC cylindrical shells with imperfection amplitude.

The load-edge shortening curves of NFRC cylindrical shells for different imperfection amplitudes are presented in Figure 5. The other parameters are *t* = 50 h and *v_f_* = 0.1. It is observed that the extreme point decreases with the increase in imperfection amplitude, which indicates that the NFRC cylindrical shell is sensitive to the initial geometric imperfection. When the imperfection amplitude is large (*μ* = 0.2 for this numerical example), the load-edge shortening curve increases monotonically without the extreme point. The variation in the critical load with the imperfection amplitude is presented in Figure 6 and the buckling modes for different imperfection amplitude are also shown. It can be observed that the critical load significantly decreases with the increasing imperfection amplitude, and decreases faster when the imperfection amplitude is small. Furthermore, the imperfection amplitude also has influence on the buckling mode.

**Figure 7:**
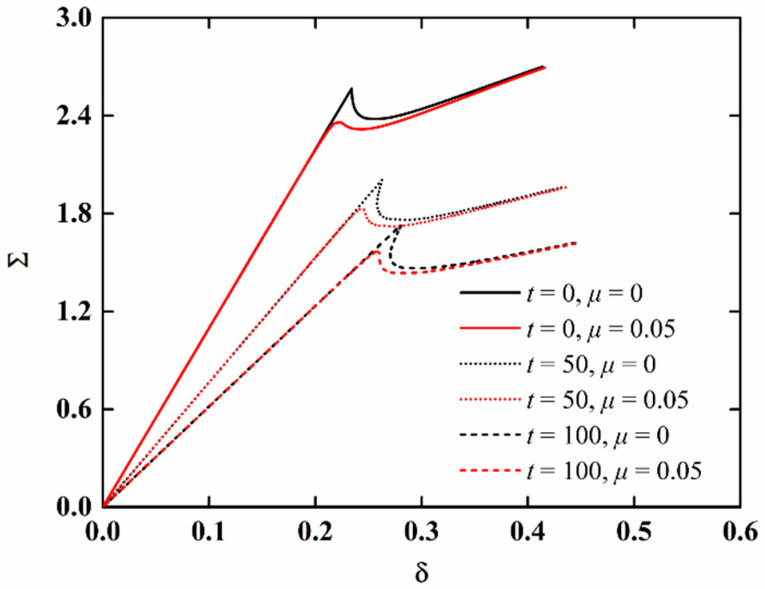
Load-edge shortening curves of imperfect NFRC cylindrical shells for different aging times.

**Figure 8:**
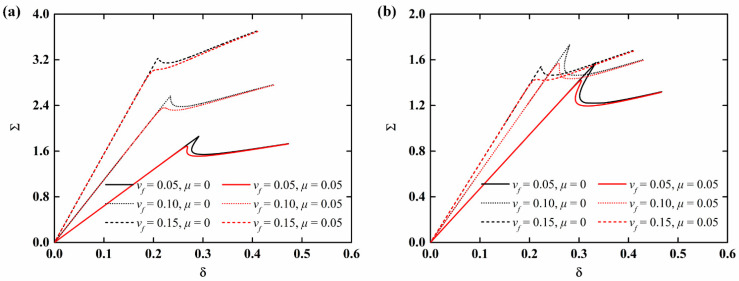
Load-edge shortening curves of imperfect NFRC cylindrical shells for different fiber contents: (**a**): *t* = 0; (**b**): *t* = 100 h.

The load-edge shortening curves of imperfect NFRC cylindrical shells for different aging time and fiber content are presented in Figure 7 and Figure 8, respectively. It can be observed that the load-end shortening curve decrease with the increase in aging time, indicating that moisture absorption and hygrothermal aging have a great influence on the stability of NFRC cylindrical shells. It can be seen from Figure 8a that when the aging time is 0 (neglecting the moisture absorption and hygrothermal aging effect), the load-end shortening curve moves up with the increase in fiber content. It is interesting, as shown in Figure 8b, that when the aging time is 100 h, the load-end shortening curve for high fiber content is lower. Here, it should be mentioned that the research aim of this paper is to qualitatively analyze the influence of moisture absorption and hygrothermal aging effect. The aging speed will be related to temperature and humidity (the coefficients in the Equations (1)–(6) will be different under different humidity and temperature), but the overall trend is similar.

**Figure 9:**
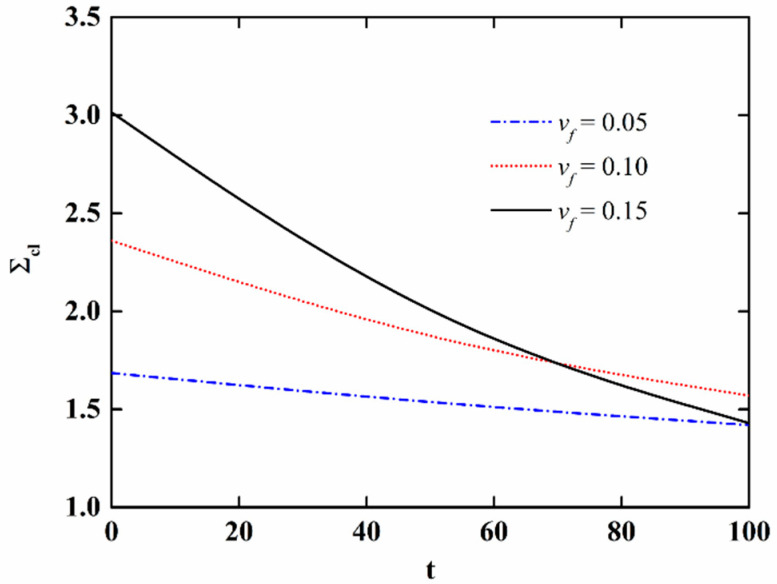
Variation in the critical load of imperfect NFRC cylindrical shells with aging time.

**Figure 10:**
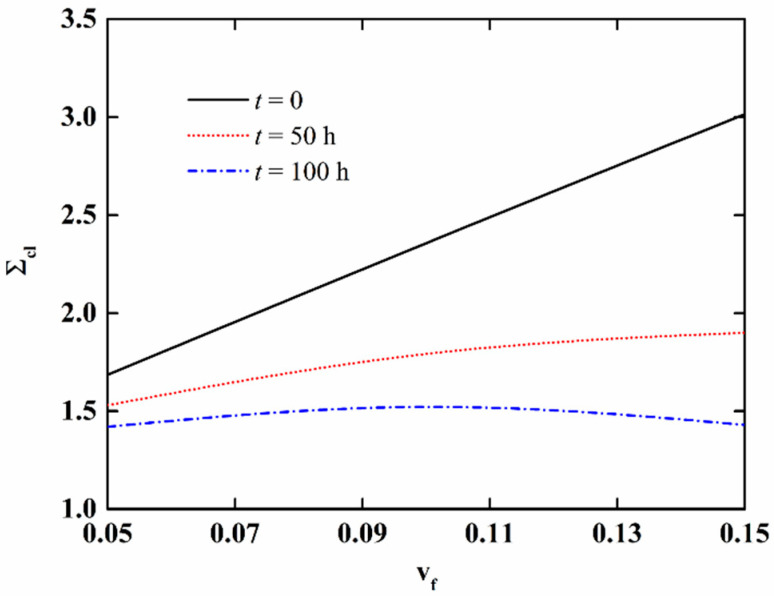
Variation in the critical load of imperfect NFRC cylindrical shells with fiber content.

**Figure 11:**
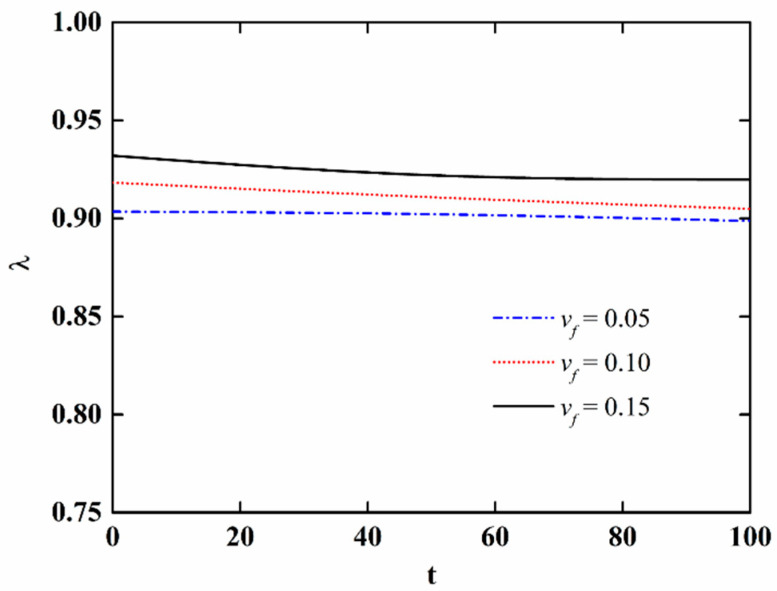
Variation in the imperfection sensitivity of imperfect NFRC cylindrical shells with aging time.

**Figure 12:**
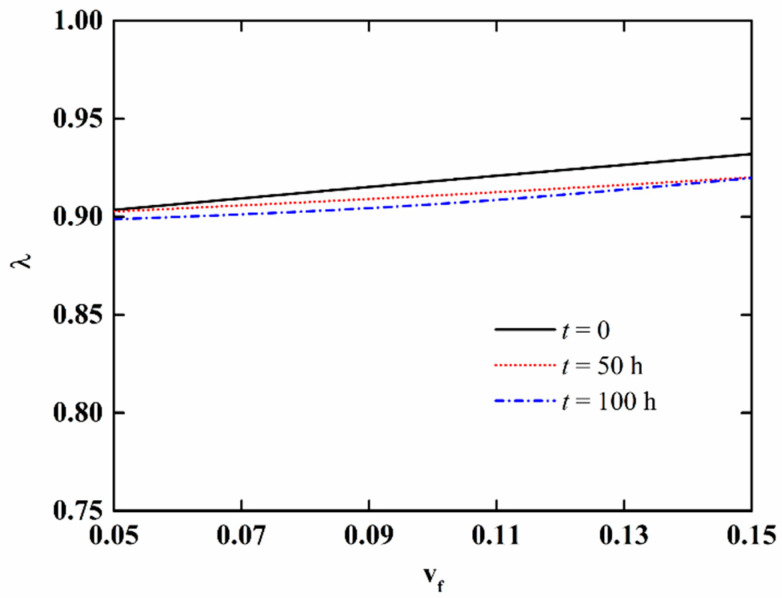
Variation in the imperfection sensitivity of imperfect NFRC cylindrical shells with fiber content.

In order to further discuss the effect of moisture absorption and hygrothermal aging on the stability of NFRC cylindrical shells, the variation in the critical load with aging time and fiber content is presented in Figure 9 and Figure 10, respectively, and the variation of the imperfection sensitivity with aging time and fiber content is presented in Figure 11 and Figure 12, respectively. The imperfection sensitivity is defined as *λ* = Σ*_cl_*/Σ*_cl_*_0_, where Σ*_cl_*_0_ and Σ*_cl_* are the critical loads for the perfect and imperfect shells, respectively. The imperfection amplitude for Figure 9, Figure 10, Figure 11 and Figure 12 is *μ* = 0.05. It is observed from Figure 9 that the critical load decreases with the increase in aging time, and the decrease is faster when the fiber content is large. It can be seen from Figure 10 that when the moisture absorption and hygrothermal aging is neglected, the critical load increases linearly with the increase in fiber content. When the aging time is *t* = 50 h, the increase in the critical load with the fiber content is very small. When the aging time is *t* = 100 h, the critical load first increases and then decreases with the increasing fiber content. It can be observed from Figure 11 and Figure 12 that the moisture absorption and hygrothermal aging has a very small influence on the imperfection sensitivity. The imperfection sensitivity increases slightly with increasing aging time and decreases slightly with increasing fiber content.

## 6. Conclusions

The nonlinear stability of natural-fiber-reinforced composite cylindrical shells with initial geometric imperfection and moisture absorption and hygrothermal aging is investigated. The nonlinear governing equations are established based on the high-order shear deformation theory. The load-edge shortening curves for different imperfection amplitudes are obtained by the Galerkin method. The effects of key influencing factors on the stability of NFRC cylindrical shells are discussed. The main conclusions are as follows: (i) NFRC cylindrical shells are sensitive to the initial geometric imperfection and small geometric imperfections will reduce the critical load; (ii) the moisture absorption and hygrothermal aging has a great influence on the stability of NFRC cylindrical shells. The critical load of NFRC cylindrical shells significantly decreases with increasing aging time; (iii) when the aging time is long, the critical load of NFRC cylindrical shells with high fiber content is even lower than those with low fiber content; (iv) the influence of moisture absorption and hygrothermal aging effect on the critical load is very obvious, while the influence on the imperfection sensitivity is very small.

## Data Availability

The data presented in this study are available upon request from the corresponding author.
